# Dual Psychological Processes Underlying Public Stigma and the Implications for Reducing Stigma

**DOI:** 10.4103/0973-1229.36546

**Published:** 2008

**Authors:** Glenn D. Reeder, John B. Pryor

**Affiliations:** * *Illinois State University*

**Keywords:** Stigma, Prejudice, Prevention, Attitudes, HIV/AIDS, Obesity, Mental illness

## Abstract

People with serious illness or disability are often burdened with social stigma that promotes a cycle of poverty via unemployment, inadequate housing and threats to mental health. Stigma may be conceptualized in terms of self-stigma (e.g., shame and lowered self-esteem) or public stigma (e.g., the general public's prejudice towards the stigmatized). This article examines two psychological processes that underlie public stigma: associative processes and rule-based processes. Associative processes are quick and relatively automatic whereas rule-based processes take longer to manifest themselves and involve deliberate thinking. Associative and rule-based thinking require different assessment instruments, follow a different time course and lead to different effects (e.g., stigma-by-association vs attributional processing that results in blame). Of greatest importance is the fact that each process may require a different stigma-prevention strategy.

## Introduction

A serious illness carries with it numerous physical, economic and mental challenges. Not only are normal life activities disrupted by physical symptoms and financial burdens that can lead to poverty but also one's sense of control and self-esteem may suffer. A new diagnosis of mental illness or human immunodeficiency virus (HIV) infection, for example, may be threatening to one's identity and the problem is compounded when significant others (such as employers and family) are perceived as reacting negatively to the diagnosis (Collins *et al*., 2000; Link *et al*., 2001). The cruel result is that social stigma – rejection in the workforce, discriminatory housing practices and unsympathetic health care workers – add to what is already a heavy burden of physical pain and suffering. In an effort to avoid personal feelings of shame and the social rejection that accompanies stigma, people often hide their illness from significant others. Such denial may even extend to avoiding diagnosis and treatment for the illness (Link and Phelan, 2006).

Goffman (1963) defined stigma as a discrediting attribute or “mark” which signals that one's identity is “spoiled” or discredited and as such, one is to be avoided in polite society. The study of stigma is exceptionally broad, posing important questions concerning economics, policy, culture and mental health (Keusch *et al*., 2006; Mawar *et al*., 2005). This article takes a psychological perspective, examining the processes that underlie public stigma.

The experience of stigma has both private and public faces (Corrigan and Penn, 1999; Jacoby *et al*., 2004). As already implied, the private face of stigma often involves feelings of grief, loss of control and anxiety on the part of the person who is stigmatized. The public face of stigma involves the general public's negative beliefs, feelings and behaviours directed toward those with a stigma (Corrigan, 2004; Corrigan and Penn, 1999). Public stigma can promote a cycle of poverty and illness in at least two ways (Baron and Salzer, 2002; Puhl and Brownell, 2003). First, employers often discriminate against those who are obese, HIV-infected or mentally ill. As one's financial resources diminish, it becomes increasingly difficult to access adequate health care. Poverty may also force one to live in sub-standard or even dangerous housing, further straining physical and mental health. Second, being poor in itself can lead to further stigmatization by the public. Self-stigma and public stigma are closely intertwined: To the extent a person believes that friends, family, landlords and employers hold stigmatizing attitudes, the person is likely to feel the pain of self-stigma. Thus, self-stigma often begins with an awareness of public stigma.

In this article, we will describe two psychological processes – one that is associative and another that is rule-based – which underlie public stigma. We will outline both the general theoretical basis for this distinction and the growing empirical evidence that supports it. Our main premise is that by increasing our understanding of the causes of public stigma, we lay the groundwork for future interventions. In turn, such interventions hold the promise of combating public stigma and thereby, disrupt the cycle of discrimination, poverty and illness.

## Public Stigma can be the Result of Two Psychological Processes

### Associative *vs* Rule-Based Processing

Consider the following examples of public stigma directed at an individual:

1. Kevin and his partner want to adopt a child with a disability. The adoption agency showed them a profile of a child with congenital HIV. When Kevin saw a cute picture of the child, he suddenly thought about homosexuality and drug abuse.
2. When checking her social networking site on the Internet, Nadia found a message from a man who said they had a lot in common and should “get together.” Nadia went to the man's Website and found her admirer to be an athletic-looking man who was shown posing next to his very obese sister. For some reason, Nadia had a bad feeling about the man.
3. Raul has been interviewing applicants for a receptionist's position in his small business. In the course of an interview, one applicant acknowledged having a history of depression. After careful consideration, Raul decided not to hire the applicant.
4. Svetlana has a friend who smoked cigarettes for 30 years and was recently diagnosed with lung cancer. For years, Svetlana had urged her friend to quit smoking. Upon hearing of her friend's diagnosis, she did not feel very sympathetic and thought to herself, “It's her own fault."

These examples deal with diverse health-related stigmas. Yet the first two examples – concerning HIV/AIDS and obesity, respectively – share a common psychological basis. They each stem from negative associations with the target individual. In Example 1, Kevin feels an aversion to a child with HIV/AIDS because he associates that illness with labels such as “homosexual” and “drug abuser.” At a factual level, Kevin knows full well that this child has never engaged in any acts relevant to these labels. But the facts are not really the issue. In Example 2, Nadia has a bad feeling about a man who is shown standing next to his obese sister. Once again, the reason for a negative reaction to the target individual is seemingly irrational. In fact, it is doubtful that Kevin and Nadia know the underlying reason for their negative feelings.

Examples 3 and 4, in contrast, describe a more thoughtful and rational process by which stigma may manifest itself. When looking to hire a receptionist for his business, Raul (in Example 3) probably is seeking a cheerful person with strong social skills. A job applicant with a history of depression might function well in other jobs within his company (for example, in accounting), but Raul may believe such a person would be a poor fit for the receptionist job. In the last example, Svetlana blames her friend for ignoring long-standing advice to stop smoking. Svetlana sees lung-cancer as an entirely controllable outcome that her friend could have avoided. These last two examples involve rule-based processing, which relies on deliberate, more or less rational information processing.

Below, we will elaborate further on the differences between associative and rule-based processing.

## Theoretical Rationale for Considering Two Psychological Processes

Research suggests that people often demonstrate considerable ambivalence to the stigmatized (Katz, 1981). For instance, although people may feel some revulsion to the physically handicapped, their actual behaviour may reflect sympathy and kindness. Consider a study in which people were asked to teach origami (paper folding) to someone in a wheel chair (Kleck, 1969). The researchers reported a discrepancy between the teachers’ verbal and nonverbal communications: on the (more controlled) verbal channel, the teachers responded quite positively to the handicapped person. Yet, on the (less controlled) nonverbal channel, the teachers showed a high level of anxiety and avoidance.

In order to accommodate such findings, social psychologists have proposed a variety of dual process models to understand prejudice and stigma (Gawronski and Bodenhausen, 2006; Pryor *et al*., 1999; Smith and DeCoster, 2000; Strack and Deutsch, 2004). A common thread in these different models is that both associative and rule-based processes are believed to shape people's reactions to a stigmatized person. Associative processes involve *automatic affective reactions*. In other words, merely being exposed to a stigmatized person immediately brings to mind negative evaluations (Fazio and Olson, 2003). The perceiver need not intend for these reactions to occur and need not invest any conscious effort to produce them. Perhaps the most important characteristic of associative evaluations is that they operate independent of the assignment of truth-value (Gawronski and Bodenhausen, 2006). In other words, negative associations can be activated in a person's mind even if the person considers those reactions to be an inaccurate characterization of a target individual (Devine, 1989). For example, upon exposure to an individual labeled as mentally ill, a perceiver may initially react with fear even though the perceiver consciously does not believe the individual is dangerous. An automatic fearful reaction of this sort may have been acquired from years of exposure to film media that depict persons with mental illness as homicidal maniacs (Wahl, 1995).

Evaluative reactions are also influenced by a second source, which we call rule-based processing. Rule-based processing involves conscious, deliberative or thoughtful reactions that rely more on “the facts”. Such deliberative processing may be triggered when a perceiver reflects on the appropriateness of his or her associative reactions to a stigmatized person (Pryor *et al*., 1999). For instance, a perceiver might feel that his or her initially fearful reaction to a person with HIV/AIDS is irrational and consequently, the person may engage in self-regulatory efforts to overcome that reaction (Monteith and Mark, 2005). In addition, the perceiver may actively consider the pros and cons of further interaction with the stigmatized person. In other words, what can be gained and what are the costs of interacting with a person with HIV/AIDS? Such processing may also rely on attributional reasoning: Is the stigmatized person held responsible for his or her adverse health outcome (Weiner, 1995)? If the person is not considered to be responsible, perceivers typically react in a sympathetic fashion. In contrast, if the person is held responsible, perceivers are more likely to react with irritation and blame the person for his or her fate. Attributional reasoning of this sort may help to explain why the victims of lung-cancer (which is typically viewed as a controllable outcome) experience greater social stigma than the victims of breast cancer (which is presumably less controllable).

### Interactions Between Associative and Rule-Based Processing

What is the nature of the interplay between associative thinking and rule-based thinking? In line with dual processing models (Gawronski and Bodhenhausen, 2006; Pryor *et al*., 2004), we assume that people usually base their evaluative reactions on their automatic associative reactions. In other words, associative processing is the default mode. But rule-based thinking may come into play if for some reason, the perceiver rejects the implications of associative processing. This sort of rejection may occur, for instance, if the perceiver believes that associative reactions violate principles of fair play (such as egalitarian beliefs or attributional considerations). For example, a health care professional may initially feel revulsion at the sight of a very obese patient. This initial reaction may give way to feelings of compassion as the professional initiates personal contact and enters into a healing relationship with the patient. In this case, the professional's conscious belief that all patients are entitled to good care triumphs over any negative associations he or she may have to obesity.

In the paragraphs below we review evidence from three lines of research that demonstrate the interplay of associative and rule-based processing. The first set of findings indicates that implicit and explicit measures of health-related stigma sometimes reveal different patterns. This divergence supports the assumption that associative processes (as measured by implicit measures) are separable from rule-based processes (as measured by explicit measures). The second set of findings demonstrates a time course to stigmatizing reactions: associative processing tends to occur prior to rule-based processing. Finally, we review evidence of “stigma-by-association” effects, whereby a target person is stigmatized simply from being associated with someone who has the stigma (for example, from being married to an obese person). The evidence will suggest that this tendency is moderated more by automatic associations than by rule-based thinking.

### Discrepancies Between Implicit and Explicit Reactions to the Stigmatized

Research on the stigma of HIV/AIDS has identified two separate types of information that contribute to prejudice (Pryor *et al*., 1999). First, information (or misinformation) about the probability of contagiousness is important. Not surprisingly, people who believe myths related to catching the HIV/AIDS from casual contact are relatively more likely to avoid persons with the disease. Beliefs about contagiousness represent rule-based concerns and suggest an explicit, rational, conscious sort of processing. But there is a second contributor to prejudice: people who are most opposed to homosexuality are particularly likely to avoid persons infected with HIV/AIDS. Aversion to homosexuality prompts aversive reactions even when the impression target is described as a little girl who contracted HIV/AIDS from a blood transfusion (Pryor *et al*., 1999). In this instance, it seems unlikely that people consciously consider the girl to be homosexual. Consequently, the contribution of anti-gay attitudes appears to be driven by affect-laden associations and suggests an implicit, somewhat irrational type of processing.

In the last ten years, social psychologists have developed separate assessment instruments for rule-based *vs* associative processing (Fazio and Olson, 2003; Gawronski and Bodenhausen, 2006; Greenwald *et al*., 1998). Rule-based processing is assessed with explicit measures that rely on standard self-report questionnaires (for example, semantic differential or Likert-type scales). The measurement of associative processing, on the other hand, relies on implicit methods such as response time measures (Greenwald *et al*., 1998) or affective priming (Payne *et al*., 2005). An important finding is that attitudes measured with explicit and implicit methods are sometimes dissociated (Gawronski and Bodenhausen, 2006). In particular, explicit measures may better predict controlled behaviour (such as what a person says to a stigmatized person), whereas implicit measures may better predict subtle or less controllable reactions (Fazio and Olson, 2003). For instance, an implicit attitude measure was a better predictor than an explicit measure of how close people would sit to an obese person (Bessenoff and Sherman, 2000).

### The Time Course of Stigma

Our analysis implies that there is a time course to associative and rule-based thinking (Pryor *et al*., 2004). People's immediate reactions to a stigmatized person are typically dominated by their associative thinking. Within a matter of seconds, however, more deliberative processing may come into play. Consider a study in which research participants received a description of a little girl who had contracted HIV/AIDS from an uncontrollable cause (a blood transfusion). When participants responded to the girl after a delay of 15 seconds, their reactions were more positive than when they responded immediately (Pryor *et al*., 1999). Apparently, immediate reactions were influenced by negative associations to HIV/AIDS (e.g., homosexuality). After a delay, reactions to the girl were adjusted in a positive direction due to rule-based processing, which relied on attributional reasoning concerning the uncontrollable cause of the stigma (Weiner, 1995). In another part of the study, participants received a description of a person who abused drugs, which represents a controllable stigma. Responses to the drug abuser were uniformly negative, irrespective of the time delay. In this case, it appears that both immediate associative reactions and delayed consideration of attributional information had similarly negative implications, resulting in the drug abuser being judged harshly in both time periods.

Another study led research participants to believe that they would don a blindfold and go on a “trust walk” (requiring physical contact) with a partner who was infected with HIV (Pryor *et al*., 2004). The partner was said to have contracted HIV from a blood transfusion he received following a car accident. Participants viewed a picture of the HIV-infected person on the left side of a computer screen and were asked to adjust the mouse curser toward or away from that person as ways to indicate a positive or negative response to the person, respectively. The mouse movement was recorded continuously over a ten second period. When analyzing this data, the researchers correlated two individual difference measures—attitudes toward homosexuality and motivation to control prejudice toward HIV-infected persons—with the position of the mouse curser at each point in time. Participants who held more negative attitudes towards homosexuality kept their distance from the HIV-infected person during the first few seconds of mouse movement. Once again, this pattern suggests that associations (to homosexuality) exert their influence immediately. The research also found that participants’ level of motivation to control their prejudice influenced mouse movements primarily during the latter half of the ten-second period. This finding suggests that such thoughtful motivation operates via a controlled, rule-based process that takes longer to manifest itself.

### Stigma-by-Association

Goffman (1963) suggested that a stigma could be like a disease that spreads from one person to the next. A person who chooses to affiliate with a member of a stigmatized group risks acquiring a *courtesy* stigma – in essence, the person is admitted into the stigmatized category. As such, both members of the stigmatized group and outsiders treat the person as if he or she is tainted with the stigma. Goffman outlined a variety of social relationships by which a courtesy stigma can be acquired including being the loyal spouse of a mental patient, the daughter of an ex-con, the parent of a handicapped child, a friend of the blind or the family of the hangman. According to Goffman, “… all share some of the discredit of the stigmatised person to whom they are related (p. 30)”.

Since Goffman's important work, phenomena of this sort have come to be called stigma-by-association. In line with Goffman's suggestion, the basis of stigma-by-association can be chosen affiliations or family ties (Corrigan and Miller, 2004). Of particular interest is recent evidence that even apparently arbitrary associations can form the basis of stigma (Hebl and Mannix, 2003). In one of these studies, research participants viewed a man who was sitting next to an obese woman while he was in the process of being interviewed for a job. Mere physical proximity to the overweight woman led participants to devalue the man's qualifications for the job.

It should be obvious by now that we view such findings as due to associative processing rather than rule-based processing. Indeed, studies within our own laboratory have both replicated and extended these results (Pryor *et al*., 2007). For example, we showed research participants a set of family photographs taken from public Internet sites. The pictures were selected and cropped such that the same man was sometimes shown next to a thin (fit) woman and sometimes shown next to an obese woman. The results indicated that the man was judged relatively less physically attractive when he was pictured with an obese woman. In addition, participants’ implicit attitudes, which assess associative processing, moderated this effect. That is, participants who scored higher on our measure of implicit prejudice (Payne *et al*., 2005) towards fat people were most likely to downgrade the attractiveness of a man pictured with an obese woman. In contrast, explicit (rule-based) attitudes regarding obesity played a lesser role in the stigma-by-association effect.

In summary, the distinction between associative processing and rule-based processing sheds light on several aspects of public stigma including discrepancies between implicit *vs* explicit attitudes, the time course of stigma and stigma-by association.

Next we will consider the implications of this analysis for interventions designed to combat stigma.

### Implications for Prevention

The devastating social and economic consequences of stigma call for a broad research agenda to inform stigma reduction programmes (Corrigan and Gelb, 2006; Heijnders and Van Der Meij, 2006; Keusch *et al*., 2006; Mawar *et al*., 2005; Link and Phelan, 2006). Research on the psychological bases of public stigma represents one important piece of this effort. In this article, we outlined evidence for two separate psychological processes that underlie public stigma: associative processes and rule-based processes. An obvious implication of our analysis is that stigma-reduction strategies need to address both of these psychological mechanisms.

Prejudice and discriminatory reactions due to associative processes typically develop over time as one is exposed to media and other culture-bound forms of communication (Gawronski and Bodenhausen, 2006; Pryor *et al*., 2004). Television and movie portrayal of mental illness, for example, can condition the general public to be irrationally fearful of individuals with mental illness (Wahl, 1995). Prevention efforts to fight such conditioning, therefore, must either (a) prevent the formation of stigmatizing images in the first place or (b) utilize conditioning principles to overcome the damage that is already done. As described below, such prevention strategies can involve mass approaches aimed at the general public (Corrigan and Gelb, 2006) or strategies that individuals can employ to de-condition themselves (Monteith and Mark, 2005).

In an effort to curtail stigmatizing images in the mass media, the National Alliance on Mental Illness (NAMI) organised *Stigma-Busters* (Corrigan and Gelb, 2006). Stigma-Busters – who now number over 15,000 advocates – watch for instances of disrespectful media representations and contact television (TV) station managers and other authorities to express their displeasure. Another mass approach utilizes public service announcements developed for radio and television in order to convey a more positive image of stigmatized persons. For example, the “*Eliminate the Barriers Initiative*” employed principles of social marketing to develop anti-stigma messages that reached millions of people (Corrigan and Gelb, 2006). The four steps of a social marketing programme include (1) problem identification (e.g., biased decisions by employers), (2) describing a target audience for an anti-stigma campaign (e.g., employers), (3) developing a technology for inducing change (e.g., designing public service announcements for TV or radio) and (4) evaluating the success of the anti-stigma campaign (Corrigan and Gelb, 2006). At the individual level, there is also reason for optimism: people who are concerned about their own automatically activated prejudices can work to overcome them (Monteith and Mark, 2005). For example, by recognizing that one is prone to stereotyping in certain situations, one can practise inhibiting those responses and consciously replacing them with more positive associations. Over time, such a self-improvement strategy has a reasonable chance of success.

Prevention strategies should also address rule-based processes, of course. In fact, the three main stigma-prevention strategies of protest, education and interpersonal contact (Corrigan and Penn, 1999) all engage rule-based-processing to some extent. Protest involves public rallies and campaigns pushing the message that discrimination against the stigmatized will not be tolerated in businesses or other public places. Such acts of protest may cause people in authority to stop and think about what they are doing, prompting less discriminatory behaviour on their part. Typically, education also aims to change an audience's conscious thoughts. Public service announcements that aim to dispel myths and replace them with facts are based on this strategy. Finally, under certain circumstances, contact with members of a stigmatized group can help to overcome stigma. NAMI's “*In Our Own Voice*” presentations, for instance, feature individuals recovering from mental illness who talk with the general public about their experiences and answer questions. Preliminary evaluations of these programmes indicate that they can produce positive changes in attitudes (Corrigan and Gelb, 2006). Although we have considered associative processes separately from rule-based processes in this discussion, some prevention efforts probably engage both processes. In particular, contact with a stigmatized group can be aimed at both increasing knowledge (a rule-based process) and creating emotional bonds (an associative process).

## Concluding Remarks

It is perhaps, appropriate that a phenomenon as important and as complex as stigma has generated a huge amount of research literature (Corrigan, 2004; Mawar *et al*., 2006). Nevertheless, many articles on the topic are highly general and have unclear relevance for the construction of successful prevention strategies. The lack of theoretical specificity in the field is also reflected in the general nature of many prevention programmes. When such broad-based programmes work, it becomes difficult to identify the reasons (i.e., the *active ingredients*) behind their success. We believe that a more targeted theoretical analysis such as that represented in our analysis of public stigma, holds the promise of promoting a more useful agenda for both basic and applied research.

### Take Home Message

In order to tear down the barriers that result from stigma, prevention efforts need to recognise the dual psychological processes – associative and rule-based – that underlie public stigma.

## Questions That This Paper Raises

1. What role does stigma play in the cycle of illness and poverty?
2. How does self-stigma depend on public stigma?
3. What two psychological processes underlie public stigma and how do these two processes interact with one another?
4. Can people learn to control their automatically activated prejudices?
5. What are the specific ingredients of successful stigma-reduction programmes and how do they address both the associative and rule-based psychological processes that underlie public stigma?

## About the Authors

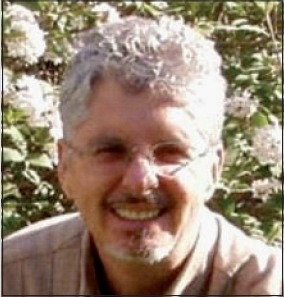

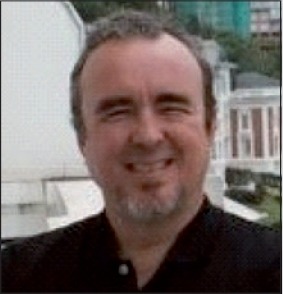





*Glenn Reeder and John Pryor were trained as social psychologists and now study the stigma that accompanies a variety of health-related conditions including HIV/AIDS, obesity and mental illness. Both authors are affiliated with the Chicago Consortium for Stigma Research and are Fellows of the American Psychological Association as well the Association for Psychological Science. As a team or separately, they have published more than 140 scientific articles, books and chapters. Glenn Reeder received his doctorate from the University of California at Santa Barbara. John Pryor received his doctorate from Princeton University.*
